# Proof of concept of a frequency-preserving and time-invariant metamaterial-based nonlinear acoustic diode

**DOI:** 10.1038/s41598-019-44843-7

**Published:** 2019-07-02

**Authors:** A. S. Gliozzi, M. Miniaci, A. O. Krushynska, B. Morvan, M. Scalerandi, N. M. Pugno, F. Bosia

**Affiliations:** 1 Department of Applied Science and Technology, Politecnico di Torino, Corso Duca degli Abruzzi 24, 10129 Torino, Italy; 2 Empa, Laboratory of Acoustics and Noise Control, Überlandstrasse 129, 8600 Dübendorf, Switzerland; 3 Computational Mechanical and Materials Engineering, Engineering and Technology institute Groningen, Faculty of Science and Engineering, University of Groningen, Groningen, 9747AG The Netherlands; 4 University of Le Havre, Laboratoire Ondes et Milieux Complexes, UMR CNRS 6294, 75 Rue Bellot, 76600 Le Havre, France; 5 Laboratory of Bio-Inspired and Graphene Nanomechanics, Department of Civil, Environmental and Mechanical Engineering, Universit di Trento, via Mesiano, 77, I-38123 Trento, Italy; 6 Department of Physics and Nanostructured Interfaces and Surfaces Centre, University of Torino, Via Pietro Giuria 1, 10125 Torino, Italy; 7 School of Engineering and Materials Science, Queen Mary University of London, Mile End Road, London, E1 4NS United Kingdom; 8 Ket-Lab, Edoardo Amaldi Foundation, via del Politecnico snc, I-00133 Roma, Italy

**Keywords:** Acoustics, Materials for devices

## Abstract

Acoustic filters and metamaterials have become essential components for elastic wave control in applications ranging from ultrasonics to noise abatement. Other devices have been designed in this field, emulating their electromagnetic counterparts. One such case is an acoustic diode or rectifier, which enables one-way wave transmission by breaking the wave equation-related reciprocity. Its achievement, however, has proved to be rather problematic, and current realizations display a number of shortcomings in terms of simplicity and versatility. Here, we present the design, fabrication and characterization of a device able to work as an acoustic diode, a switch and a transistor-like apparatus, exploiting symmetry-breaking nonlinear effects like harmonic generation and wave mixing, and the filtering capabilities of metamaterials. This device presents several advantages compared with previous acoustic diode realizations, including versatility, time invariance, frequency preserving characteristics and switchability. We numerically evaluate its efficiency and demonstrate its feasibility in a preliminary experimental realization. This work may provide new opportunities for the practical realization of structural components with one-way wave propagation properties.

## Introduction

In acoustics as well as in electromagnetism, the invariance of the wave equation under time inversion leads to the fundamental property of reciprocity, i.e. symmetrical wave propagation between two points in space, independently of which is the source and which is the receiver. This has been widely exploited in the so-called time-reversal technique, which enables to focus on a source or scatterer by time-reversing and retransmitting the signal recorded by an array of transducers^1^. However, reciprocity is not necessarily desirable in all cases, especially when the goal is to isolate a source from its echos. Removal of unwanted reflections could indeed find numerous applications, such as acoustic one-way mirrors to prevent an ultrasound source from being disturbed by reflected waves^2,3^, unidirectional sonic barriers to block environmental noise in a predefined direction^4^, control of acoustic energy transmission in medical applications using focused ultrasound^5^, and energy harvesting^6^. To achieve this, researchers in the field of acoustics and ultrasonics have drawn inspiration from electromagnetism, in the quest for a simple and efficient realization of an Acoustic Diode (AD) or rectifier. However, as illustrated by Maznev *et al*.^7^, linear elastic systems cannot be exploited to create ADs or isolators because they do not violate the reciprocity principle, so that the symmetry needs to be broken, for instance by periodically varying the elastic properties in space and time or by means of the introduction of nonlinearity coupled with some other mechanism (e.g. attenuation)^7^.

With this in mind, a periodical variation of elastic properties in space and time has been exploited in theoretical and numerical studies of one-dimensional systems described by the discrete nonlinear Schrödinger equation with spatially varying coefficients embedded in a linear lattice^8^, or in continuous elastic systems with periodically-modulated elastic properties in space and time^9^, or in non-reciprocal active acoustic metamaterials^10^. In other works, the introduction of nonlinearity has been the adopted strategy, such as in the 1-D design of a “superlattice” structure coupled with a nonlinear elastic medium^2^, later realized experimentally using a contrast agent microbubble suspension to generate the nonlinearity^3^, converting energy from the fundamental frequency to higher harmonics^7^. Since then, several experimental realizations of ADs or rectifiers based on different mechanisms have been achieved. In one case, unidirectional transmission was obtained through mode conversion, using a sonic crystal, rather than elastic nonlinearity^11^. In another, a mechanical energy switch and transistor are implemented by exploiting nonlinear dynamical effects of a granular crystal chain^12^. To break the transmission symmetry, another study proposed to use a subwavelength acoustic resonant ring cavity filled with a circulating fluid, splitting the degenerate azimuthal resonant modes, in analogy with the Zeeman effect in electromagnetism^13^. In another realization, a thin brass plate with single-sided periodical gratings immersed in water was shown to provide unidirectional transmission in a broad frequency range^14^. Finally, a passive multi-port structure with asymmetric sound transmission between neighbouring ports was presented^15^. Comprehensive reviews of these and other approaches can be found in^7,16^, in the latter with special reference to information processing in phononic computing, while the optimization of a rectifier efficiency in periodic mass–spring lattices is discussed in^17^.

Many of these approaches are based on designing periodic structures, mainly phononic crystals and elastic metamaterials, which have attracted much attention for their wave manipulation capabilities, including negative refraction^18^, frequency Band Gap (BG) formation^19–21^, wave filtering or focusing^22–25^, scattering-free propagation^26^ and acoustic cloaking^27^. Recent studies have shown how structural instabilities induced in “static” mechanical metamaterials can be exploited to achieve highly nonlinear dynamic response that can be tailored to requirements^28,29^ and how weakly nonlinear monoatomic lattice chains can provide active control on elastic waves in phononic crystals^30^. These or other approaches can be exploited to generate the type of nonlinearity required to violate spatial reciprocity in elastic wave propagation^31^. On the other hand, phononic crystals and metamaterials are ideal candidates to efficiently realise large BGs^32,33^ or to concentrate energy into selected frequency ranges^25,34^.

In this paper, we propose the realization of an AD, based on the use of linear phononic crystals and elastic metamaterials, embedded between elastic nonlinear regions. The novelty of the device is that it is simultaneously time–invariant (in the sense that its physical properties are not modified externally from the forward to the backward propagation direction^35^) and frequency preserving. Furthermore, besides its functionality as a diode, the device can be activated or deactivated at will for other applications, transforming it into a switch with the additional possibility to tune the amplitude of the output signal. These characteristics are in general not concurrently present in other AD designs that exploit nonlinearity to break the propagation symmetry and to transfer energy from the fundamental to the harmonics, with a frequency variation from input to output. The originality of our approach also resides in the exploitation of the combined effects of two different features of nonlinear elastic wave propagation, i.e. higher order harmonic generation and wave mixing, which allow to preserve the operating frequency occuring in two different zones separated by the periodic (filtering) structure. We recall that wave mixing occurs when two longitudinal waves propagating through a nonlinear elastic zone interact and generate another longitudinal wave with a frequency given by the difference (and sum) of the frequencies of the two original waves.

## Working Principle

**Figure 1:**
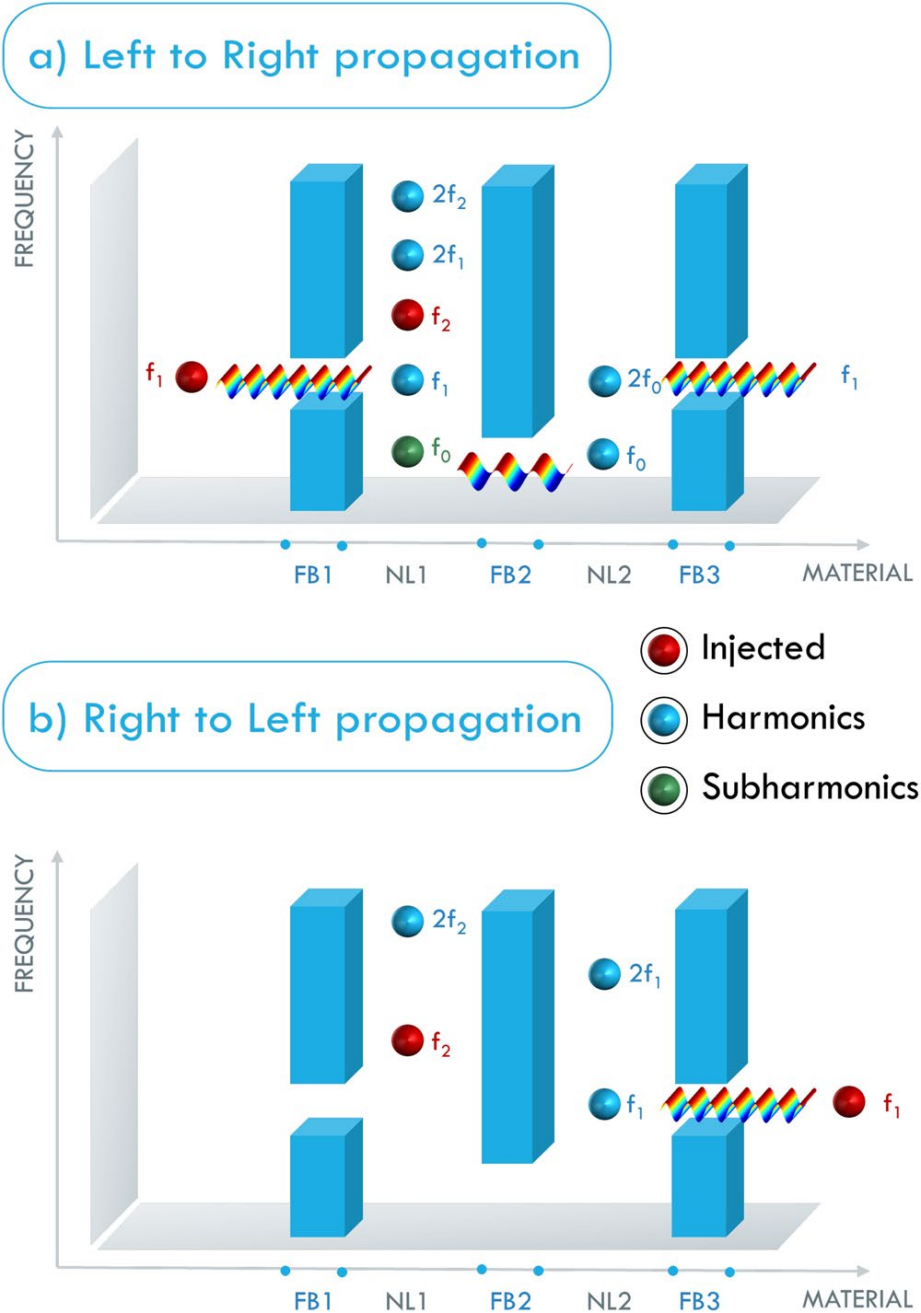
Schematic representation of the basic concept of the proposed AD for (**a**) left to right and (**b**) right to left propagation, respectively. *f*_1_ and ${f}_{2}=\frac{3}{2}{f}_{1}$ are the injected wave components, while ${f}_{0}=\frac{1}{2}{f}_{1}$ is generated by wave mixing. The blue barriers represent the frequency BGs, between them are the nonlinear cavities (NL1 and NL2) where harmonic generation and wave mixing take place. The overall effect of the device is to trasmit *f*_1_ from left to right, but not from right to left.

The working principle of the AD proposed in this study is illustrated in Fig. 1 and can be described as follows:

(i). *Propagation from left to right (LtR*, Fig. 1a): an input signal is injected (from *S*_1_) into the device where it encounters a passband filter FB1 that selects a range of frequencies around *f*_1_. These waves then travel through a first nonlinear elastic zone, named NL1, where a second frequency ${f}_{2}=\frac{3}{2}{f}_{1}$ can be injected from the source *S*_2_. In this case, the presence of nonlinearity generates higher harmonics and the sum and difference frequencies (wave mixing), including ${f}_{2}-{f}_{1}={f}_{0}=\frac{{f}_{1}}{2}$, which is a subharmonic of *f*_1_. The next portion of the device, FB2, is a low-pass filter, which eliminates frequencies above *f*_0_, and a second nonlinear zone, NL2, where the second harmonic *f*_1_ = 2*f*_0_ is generated. Finally, another passband filter (FB3) filters out *f*_0_ and the harmonics higher than *f*_1_, giving an overall output signal *f*_1_ at the same frequency of the input.
(ii). *Propagation from right to left (RtL*, Fig. 1b*):* in this case, the input signal at *f*_1_ travels through FB3 and through NL2 where higher harmonics are generated (but not *f*_0_), and where no wave mixing process takes place (this breaks spatial reciprocity). The next portion of the device, FB2, filters out the full signal, so that no signal propagates through NL1 and FB1, generating no output from the device.

Notice that the source *S*_2_ is present both in the forward and in the backward propagation (in this sense the AD is time invariant in its physical characteristics) and its role is to break spatial symmetry in the device. This mechanism allows us to overcome some of the difficulties in the practical realization encountered in other theoretical works that propose frequency-preserving ADs^36–38^. The present model/configuration has been conceived for monochromatic inputs, as usually done for nonlinearity-based ADs. More complicated designs can be considered by imposing a non-monochromatic wave injected by the source *S*_2_. However, this is beyond the scope of this work.

## Results

**Figure 2:**
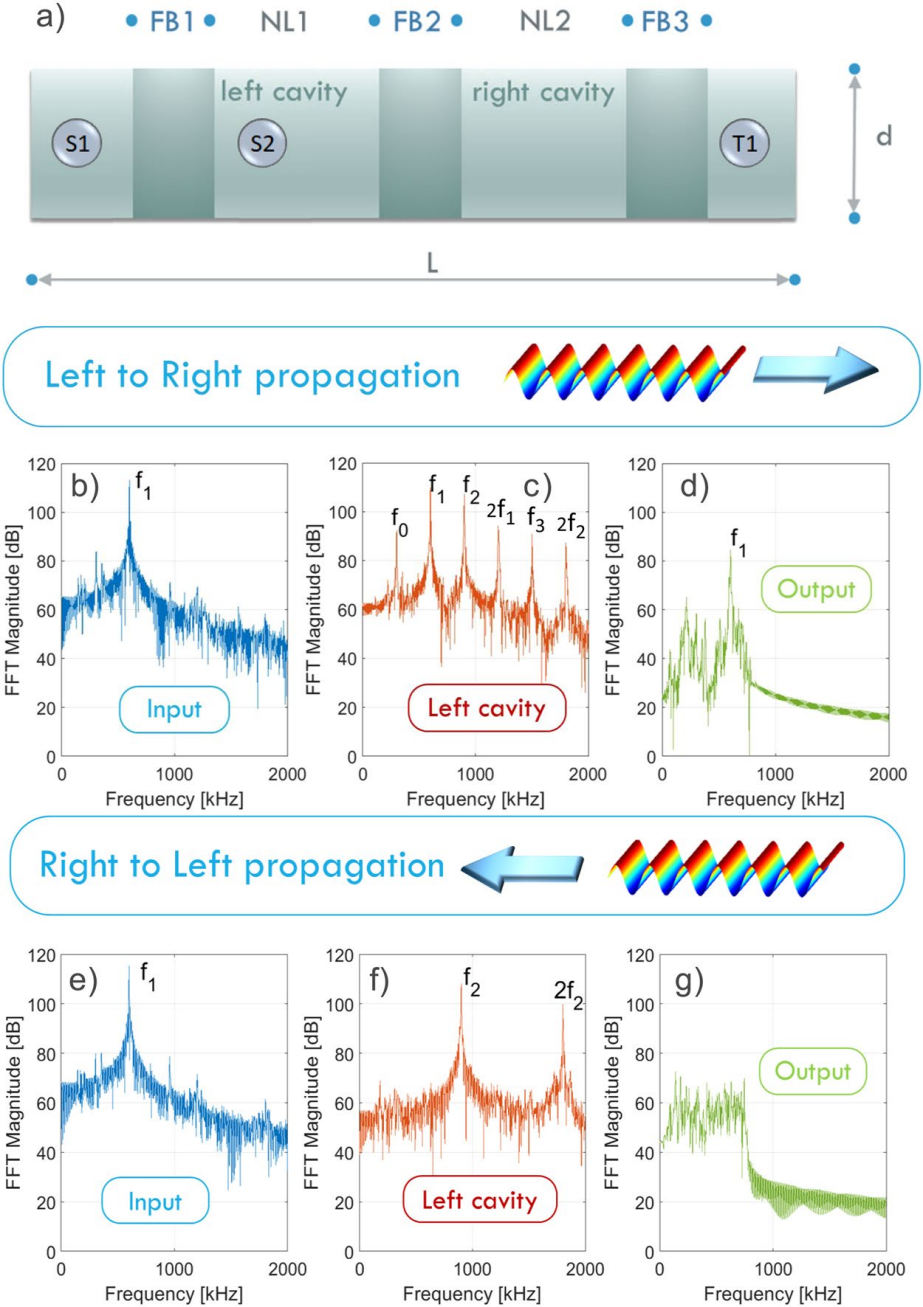
Numerical simulations. Schematic of the considered sample (**a**); Fast Fourier Transforms of the signal recorded in the input (**b**,**e**), in the first cavity on the left (**c**,**f**) and at the output (**d**,**g**), for the two propagation directions (LtR and RtL in the first and second rows, respectively).

**Figure 3:**
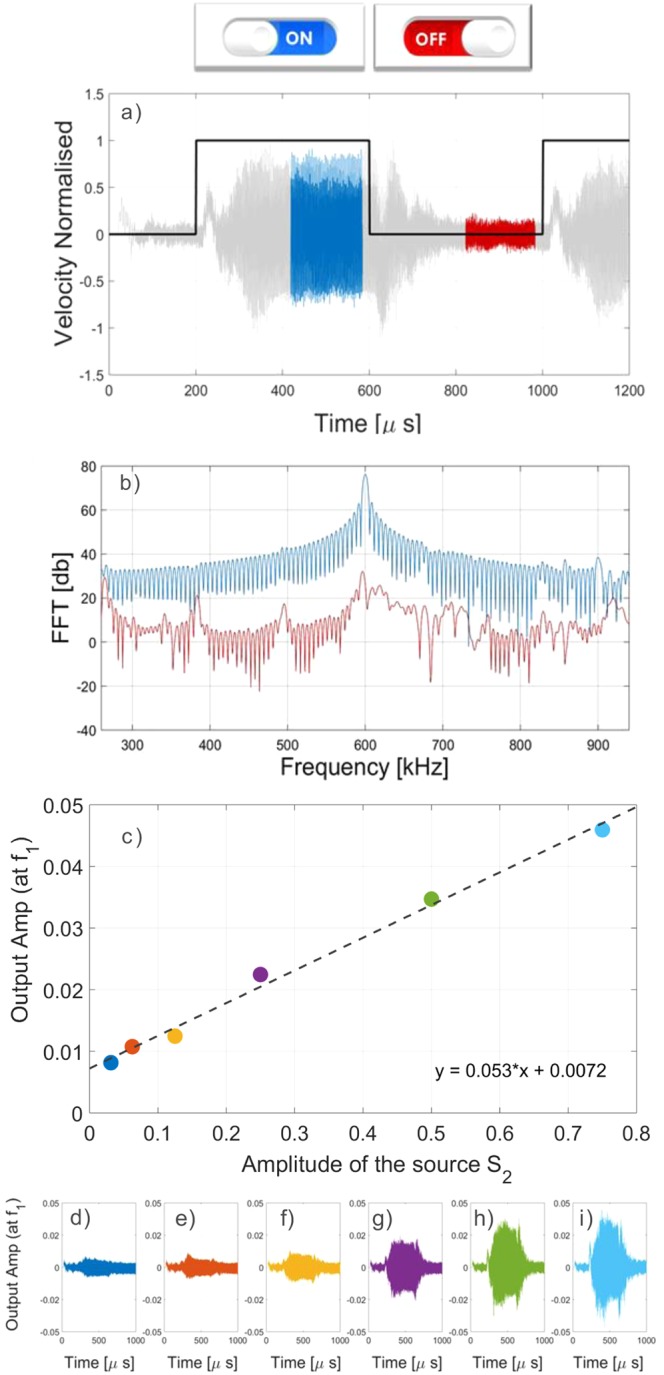
Acoustic on-off and amplitude-tunable switch. By switching on/off the source *S*_2_, the wave generated by *S*_1_ can/cannot propagate through the device. This is visible in numerical simulations in both the (**a**) time and (**b**) frequency domain. The FFT performed over different time windows (highlighted with different colors in subplot (**a**)) shows the different frequency content of the propagating wave. Results relative to a tuning-amplitude switch are shown in (**c**): the output amplitude linearly increases as a function of the pump amplitude *S*_2_, for constant input amplitude, *S*_1_ (the equation of the fitting function is also reported); (**d**–**i**) Corresponding outputs signals.

**Figure 4:**
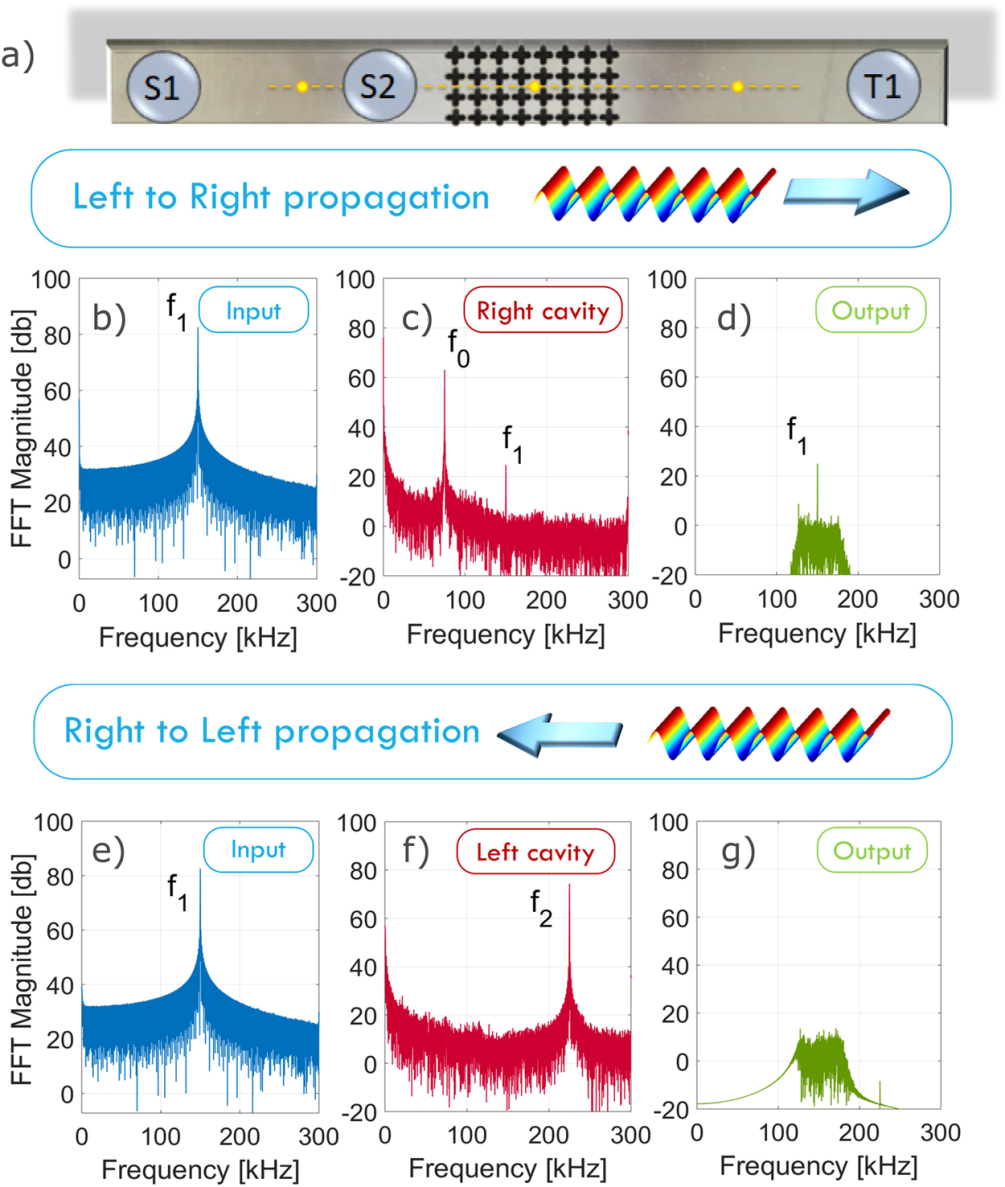
Experimental set up (**a**) and results for LtR (**b**–**d**) and RtL (**e**–**g**) propagation. In the first and second columns, the FFT of the injected signal (**b**,**e**) and of the output (**c**,**f**) in the right and left cavities, are reported, respectively. The third column shows the simulated effect on the FFT of the output of the phononic barriers in the full device.

In this work, we exploited the filtering properties of two different types of phononic crystals, described in the Methods Section and Supplemental Material, for the Filtering Barrier regions of the device. To verify the feasibility and functionality of the device, we first performed wide-ranging simulations of its general characteristics (Figs 2 and 3) and then verified results in a preliminary experimental realization (Fig. 4).

### Numerical simulations

Figure 2a shows a schematic of the sample considered in the simulations described in the Methods section. The excitation signal (a sinusoidal wave) is uniformly applied at the left boundary of FB1 (for LtR propagation) or at the right boundary of FB3 (for RtL propagation). We assume reflecting conditions at the boundaries that are free from excitation.

With this configuration, we perform wave propagation simulations to demonstrate the feasibility of the AD. For the LtR (RtL) propagation, we inject a monochromatic wave of frequency *f*_1_ = 600 kHz on the left (right) side of the device and the corresponding *f*_2_ = 900 kHz in the left cavity. The output signal is recorded on the left (right) side of the sample (T1 in Fig. 2a). Figure 2 shows the Fast Fourier Transform (FFT) of the signals for LtR (b–d) and RtL (e–g), respectively. The signals are recorded at the input of the device (b,e), in the first cavity on the left (c,f) and at the output (d,g). While *f*_1_ propagates from LtR, no signal is detected at the receiver when the propagation is in the other direction. In the output of the RtL propagation, only noise is present and no energy transmission is detectable. This proves the very good performance of the acoustic diode device. The difference between the two cases (reported in the upper and lower parts of Fig. 2(b–g), respectively) lies in the generation in the left cavity of the frequency *f*_0_, which is the only component that can propagate from NL1 to NL2. In the left cavity (Fig. 2c) we observe the presence of the harmonics of the foundamental frequency generated by the two sources and the two other frequencies *f*_0_ = *f*_2_ − *f*_1_ and *f*_3_ = *f*_1_ + *f*_2_, due to the nonlinear frequency mixing process.

Although any mechanism able to generate sub-harmonics^39,40^ of *f*_1_ can be appropriate, the mechanism based on wave mixing adopted here to generate *f*_0_ has several advantages. The first is that wave mixing is an extremely efficient way to produce sub-harmonics and no threshold mechanism appears to be present. Moreover, the source *S*_2_ can be tuned in order to decrease or increase the amplitude of the *f*_0_ component, and in the limit case to suppress it. Thus, the device can be used as an *on-off* or an *amplitude-tuning* switch. Two different simulations are presented to demonstrate these applications.

In the first case, the source *S*_2_ (the pump) is switched on/off at regular time intervals and the corresponding output recorded (Fig. 3a). It is clear that the signal is prevented from propagating when the source *S*_2_ is switched off. This is also evident in the FFT analysis performed by windowing the time signal for the two different cases (*S*_2_ on/off in Fig. 3b). This demonstrates the use of the AD as an on/off switch.

The same numerical experiment is then repeated at increasing amplitude of the pump (*S*_2_) while keeping the amplitude of *S*_1_ fixed. Since the amplitude of the *f*_0_ component (the subharmonic of the input) is proportional to the product of the two mixed frequency amplitudes, it is possible to vary the output signal amplitude by tuning the amplitude of the pump (*S*_2_, in this case), as shown in Fig. 3(c–i). This generates the possibility to realize a switch with a variable amplitude output (i.e. a transistor-like apparatus). Moreover, from a theoretical point of view, this provides the possibility of considerably increasing the efficiency of the device by pumping energy from *S*_2_ and increasing the output amplitude at will. Indeed, despite the presence of an additional nonlinear process, theoretical considerations and numerical simulations indicate that the efficiency of the AD proposed could be greatly increased by playing with the nonlinear parameters and by tuning the amplitude of the pumping energy, as discussed in the Supplemental Material. From a practical point of view, a large amplification at the pump transducer (*S*_2_) may be limited by spurious nonlinear effects and by the large amount of energy required. Nevertheless, considering reasonable experimental limitations, we could still achieve an efficiency of the order of a few percent, which is comparable to the results obtained with other nonlinear-based ADs involving a single nonlinear process^3,41^. Furthermore, more efficient systems to generate nonlinearity could also be employed to further improve the efficiency of the device, e.g. that presented in ref.^42^, so that the performance can be enhanced by two orders of magnitude.

### Experimental realization

The discussed design of the AD is quite general and can be realized with different nonlinearity types, filtering characteristics or optimized properties. We demonstrate its feasibility through the experimental realization of a prototype, shown in Fig. 4a, representing the central part of the device, which is responsible for the breaking of reciprocity. The experimental procedure is discussed in detail in the Methods section. Two source trandsucers (*S*_1_ and *S*_2_) are placed in the first nonlinear cavity (NL1), and a target transducer (*T*_2_) in the second nonlinear cavity NL2. In this case, to exploit the phononic barrier characteristics, we set *f*_1_ = 150 kHz and *f*_2_ = 225 kHz.

Figure 4(b–d) illustrate LtR propagation, as detected by the laser vibrometer, while Fig. 4(e–g) refer to RtL propagation. For the LtR propagation, *S*_1_ is placed in NL1 and *T*_1_ in NL2, while for LtR, they are inverted, leaving the position of *S*_2_ unchanged. The FFT of the input signal injected at the source *S*_1_ and of the output are shown in the first and in the second columns, respectively. The filtering action of FB1 and FB3 in the complete device, i.e. the effect of the phononic barriers, is simulated here in post-processing by imposing a numerical band-pass filter (centered around *f*_1_ = 150 kHz) on the output signals (Fig. 4d,g). Despite the relatively small amplitudes, the symmetry breaking in the wave propagation for the frequency *f*_1_ is evident in the two considered propagation directions. The difference in the output obtained in the left and right propagation demonstrates the functionality of the AD.

## Conclusions

In summary, we have presented numerical and experimental results demonstrating the feasibility of an acoustic diode based on alternating nonlinear elastic and metamaterial frequency-filtering regions, with time-invariant, frequency preserving characteristics. The design concept is on the one hand rather simple, since it is based on the sequential repetition of only two basic building blocks, i.e. nonlinear cavities and metamaterial-based frequency filters (low-pass and bandpass); on the other hand, it is sufficiently general to allow flexibility in its realization, involving different combinations of nonlinearity and BG mechanisms, and the use of phononic crystals or resonant metamaterials provides the opportunity to tune and scale results to the desired device sizes and frequency ranges. Additionally, the adoption of an input monochromatic driving signal allows the adaptation of the concept to different types of devices, such as switches or transistors, which can be exploited in practical applications in the field of acoustics or ultrasonics^43^. These can potentially be coupled and integrated with recently introduced metamaterial-based sensors for damage detection and localization^25^ or for other advanced signal manipulation purposes, including in quantum acoustodynamics^44^. On the other hand, AD-based devices could also be exploited for energy harvesting, exploiting their unidirectional transmission characteristics for elastic wave energy trapping. Improvements in the nonlinear elasticity generation mechanisms are currently under study to provide improved device stability and efficiency, potentially leading to its integration in advanced apparatuses requiring one-way transmission.

## Methods

### Numerical calculations

In numerical simulations, we model the device as an Aluminum plate with mass density *ρ*_1_ = 2700 kg/m^3^, Young modulus *E* = 70 GPa, and Poisson ratio *ν* = 0.33 and in-plane dimensions *L* = 105 mm and *d* = 6.6 mm (Fig. 2a). The core of the device, in which reciprocity is broken, is composed by two nonlinear zones (NL1 and NL2 in Fig. 2a), separated by a metamaterial (FB2).

The nonlinear sections NL1 and NL2 are realized by considering a zone of diffuse nonlinearity, and the numerical nonlinear parameters are set in order to produce about 10% of harmonics and subharmonics. These two nonlinear zones are placed between two filters made of metamaterials or phononic crystals, which confine the frequency components of the wavefield falling in their BGs, creating a sort of resonant cavity (also denominated left and right cavities in the following). The dimensions of these regions and of the nonlinear elements can be tailored to enhance the desired frequencies through resonance effects (*f*_0_ in the left, and *f*_1_ in the right cavity). A nonclassical nonlinear model^45–47^, implemented using a Preisach-Mayergoyz^48^ space representation, is adopted to simulate the nonlinear elastic response of these zones.

The structure of each metamaterial/phononic crystal part (FB1-FB3) is described in detail in the Supplementary Material together with its dispersion characteristics. The scalability of the results is guaranteed by the fact that the geometry of the constituent elements can easily be tuned to shift the pass bands to the desired frequencies.

### Experimental

For the experimental verification of the proposed working principle, we use a 380 × 40 × 6 mm^3^ aluminium plate (*ρ* = 2700 kg/m^3^, *E* = 70 GPa and *ν* = 0.33) with a phononic crystal region representing the filtering barrier (FB2 in Fig. 1). The phononic crystal is located between two regions that represent the left (with NL1) and right (with NL2) cavities in Fig. 1. FB2 consists of a 2D array of 4 × 8 cross-like cavities, fabricated using waterjet cutting, with a lattice parameter of *a* = 10 mm (see Supplemental Material for geometrical details). Dimensions have been designed so as to suppress frequencies from 124  kHz to 175 kHz and 191 kHz to 236 kHz, in the propagation from one cavity to the other (see Supplemental Material for further details). It follows that the working frequency of this AD is *f*_1_ = 150 kHz, while the pump *S*_2_ needs to be set at a frequency *f*_2_ = 225 kHz. In this simplified realization, in the LtR propagation, the two sources (*S*_1_ and *S*_2_) are located in the same cavity on the left, while the receiver (*T*_1_) is situated in the right cavity, as shown in Fig. 4a. The nonlinearity is generated in the two cavities, by superposing onto the plate a small object coupled with a drop of water^25^. The clapping of the surfaces, due to the action of the elastic wavefield propagating in the plate gives rise to typical nonlinear effects (i.e. harmonics and wave mixing). However, this method to generate nonlinearity is not sufficiently stable to allow tunability of the output amplitude through the *S*_2_ input amplitude, so that the transistor-like switch functionality cannot be implemented. However, it serves the purpose of demonstrating the diode functionality in a proof-of-concept experiment.

In the experiments, the emitting piezoelectric contact transducer was connected to an arbitrary waveform generator (Agilent 33500B) through a 50 dB linear amplifier (FLC Electronics A400). The receiving transducer/laser interferometer was connected to an oscilloscope (Agilent Infiniium DSO9024H) for data acquisition.

## Supplementary information

Supplemental Material
