# Supplementary material for: Proof of concept of a frequency-preserving and time-invariant metamaterial-based nonlinear acoustic diode
Source: Sci Rep. 2019 Jul 2;9:9560. doi: 10.1038/s41598-019-44843-7 (PMC6606629; doi:10.1038/s41598-019-44843-7)
Supplement: Supplementary file 1 — Supplemental Material [file 41598_2019_44843_MOESM1_ESM.pdf]

# Supplemental Material for "A frequency-preserving and time-invariant metamaterial-based nonlinear acoustic diode"

A. S. Gliozzi,[1,*] M. Miniaci,[2,†] A. O. Krushynska,[3] B. Morvan,[4] M. Scalerandi,[1] N. M. Pugno,[5,‡] and F. Bosia[6]

[1]*Department of Applied Science and Technology, Politecnico di Torino, Corso Duca degli Abruzzi 24, 10129 Torino, Italy*

[2]*Enpa, Laboratory of Acoustics and Noise Control, Überlandstrasse 129, 8600 Dübendorf, Switzerland*

[3]*Engineering and Technology Institute Groningen, Faculty of Science and Engineering, University of Groningen, Groningen 9747AG, the Netherlands*

[4]*University of Le Havre, Laboratoire Ondes et Milieux Complexes, UMR CNRS 6294, 75 Rue Bellot, 76600 Le Havre, France*

[5]*Laboratory of Bio-Inspired and Graphene Nanomechanics, Department of Civil, Environmental and Mechanical Engineering, Università di Trento, via Mesiano, 77, I-38123 Trento, Italy*

[6]*Department of Physics and Nanostructured Interfaces and Surfaces Centre, University of Torino, Via Pietro Giuria 1, 10125 Torino, Italy*

(Dated: May 8, 2019)

[*]Electronic address: antonio.gliozzi@polito.it

[†]Also at: University of Le Havre, Laboratoire Ondes et Milieux Complexes, UMR CNRS 6294, 75 Rue Bellot, 76600 Le Havre, France

[‡]Also at: School of Engineering and Materials Science, Queen Mary University of London, Mile End Road, London E1 4NS, United Kingdom and Ket-Lab, Edoardo Amaldi Foundation, via del Politecnico snc, I-00133 Roma, Italy

## I. NUMERICAL MODELS

We first provide a detailed geometrical description of the filtering barriers FBi (with $i \in [1, 2, 3]$) presented in Fig. 1 of the main text, along with their dynamical behaviour in terms of band gaps (BGs) and pass bands.

### A. Design of the filtering barrier 1 (FB1) and filtering barrier 3 (FB3)

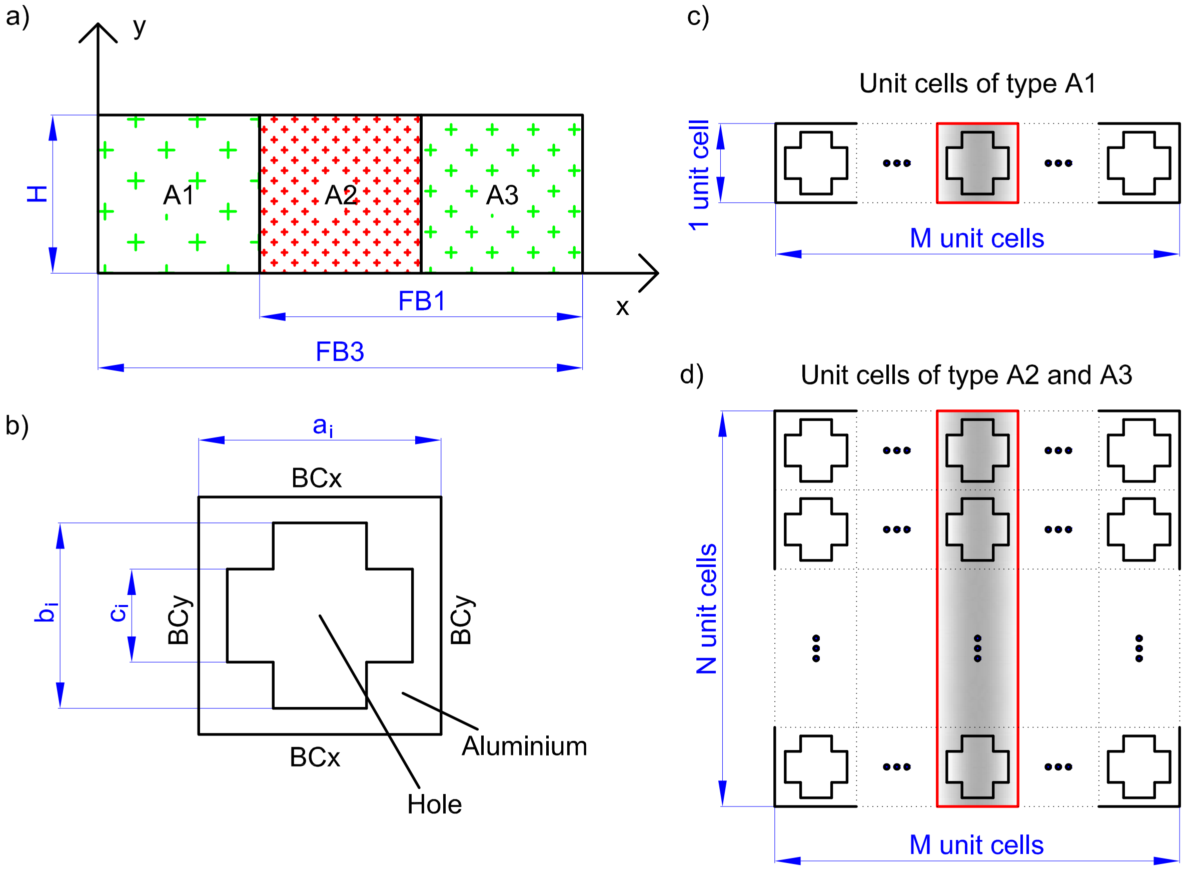


FIG. SM1: (a) Schematic representation of the FB1 and FB3 regions made of (b) periodic cross-like holes in an aluminium matrix and (c,d) their geometrical arrangements in $x$ and $y$ directions within the $A1$, $A2$ and $A3$ regions. The geometrical parameters $a_i$, $b_i$, $c_i$ of the unit cells are reported in Table I and the mechanical properties given in the text.

Fig. SM1a shows a schematic representation of the filtering barrier 1 (FB1) and filtering barrier 3 (FB3) regions.

TABLE I: Geometrical parameters for the unit cells presented in regions $A1$, $A2$ and $A3$ shown in Fig. SM1.

| Geometrical parameter | Value [mm] | | |
|---|---|---|---|
| | case $i = 1$ | case $i = 2$ | case $i = 3$ |
| $a_i$ | 6.60 | 0.66 | 1.20 |
| $b_i$ | $0.78 \cdot a_1$ | $0.90 \cdot a_2$ | $0.95 \cdot a_3$ |
| $c_i$ | $0.18 \cdot a_1$ | $0.20 \cdot a_2$ | $0.20 \cdot a_3$ |

FB1 is made of two phononic regions, namely $A2$ and $A3$ whereas FB3 is made of three phononic regions, namely $A1$, $A2$ and $A3$. All these regions are characterized by the presence of periodic cross-like holes into an aluminium matrix (Fig. SM1b) with the following mechanical properties: $\rho = 2700$ kg/m$^3$, $E = 70$ GPa and $\nu = 0.33$. The geometrical parameters $a_i$, $b_i$ and $c_i$ of the unit cells are reported in Table I and are chosen so as to provide proper filtering characteristics in specific frequency ranges (see Fig. 1 of the main text and Fig. SM3 of the next section). The number of unit cells chosen along the $y$-direction ($N$ in Figs. SM1c,d) derives from the choice of adopting the same height $H = 6.6$ mm for the entire device, whereas the number of repeated unit cells along the $x$-direction ($M$ in Figs. SM1c,d)) is dictated by the need to achieve an attenuation of the wave amplitude within the BG frequencies of at least one order of magnitude [1].

### B. Design of the filtering barrier 2 (FB2)

Fig. SM2 shows a schematic representation of the filtering barrier 2 (FB2). FB2 is made of locally resonant structures using heavy coated inclusions (highlighted in red in the figure) connected by thin ligaments. This choice is principally due to the type of filtering action required by FB2, i.e. a low pass band filter. The dispersion diagram originated by a single locally resonant structure adapts well to this need (see Fig. SM4 in the next section), contrary to the cases of FB1 and FB2, where pass bands within a lower frequency and a higher frequency filtering regions are required. Furthermore, this choice allows to minimize the size of the device, compared to using phononic crystals for the FB2 region, too. The resonators and ligament dimensions are reported in Table II and are tailored to provide

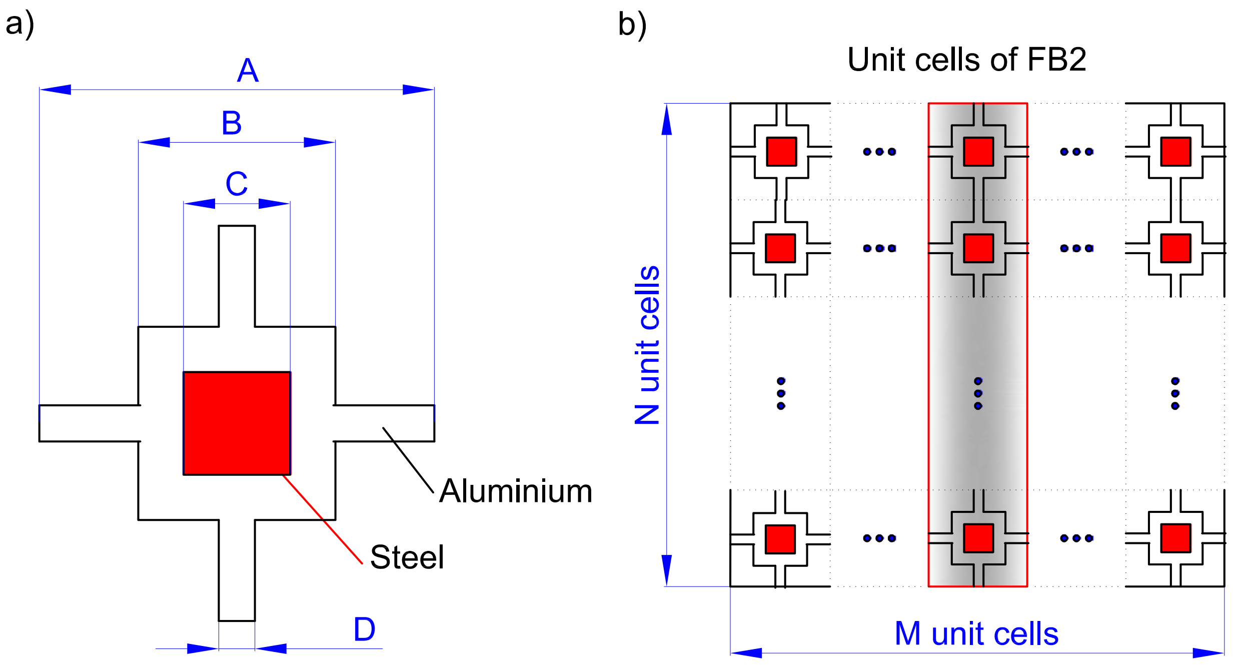


FIG. SM2: Schematic representation of the FB2 region made of (a) a locally resonant structures using heavy coated inclusions connected by thin ligaments. The resonators are square in shape and their dimensions are tailored to define the required properties of the barrier. The geometrical parameters of the unit cell are reported in Table II ($\rho = 2700$ kg/m$^3$, $E = 70$ GPa and $\nu = 0.33$) while the internal core made of steel has the following properties: $\rho = 7784\,\mathrm{kg/m^3}$, $E = 207\,\mathrm{GPa}$ and $\nu = 0.30$.

proper filtering abilities in specific frequency ranges (see Fig. 1 of the main text and Fig. SM4 of the next section). The geometrical parameters of the unit cell are reported in Table II. The matrix is made of aluminium ($\rho = 2700$ kg/m$^3$, $E = 70$ GPa and $\nu = 0.33$) while the internal core made of steel with the following properties: $\rho = 7784$ kg/m$^3$, $E = 207$ GPa and $\nu = 0.30$.

As in the previous case, the number of unit cells chosen along the $y$-direction ($N$ in Figs. SM2b) derives from the choice of adopting the same height $H = 6.6$ mm for the entire the device, whereas the number of repeated unit cells along the $x$-direction ($M$ in Figs. SM2b)) is dictated by the need to achieve an attenuation of the wave amplitude within the BG frequencies of at least one order of magnitude [1].

TABLE II: Geometrical parameters for the unit cell presented in Fig. SM2a.

| Geometrical parameter | Value [mm] |
|---|---|
| A | 1.65 |
| B | 1.15 |
| C | 0.82 |
| D | 0.07 |

## II. NUMERICAL RESULTS

In this section, dispersion diagrams for the regions described in the previous section are computed by applying the Bloch-Floquet theory. This allowed to consider only one so-called unit cell, i.e. the smallest representative geometry for computing the dispersion diagram [2]. The numerical models are implemented in a 2D-plane strain assumption. Cells are meshed by means of 4-node quadrilateral elements of maximum size $L_{FE} = 0.1$ mm in order to provide accurate eigensolutions up to the maximum frequency of interest of 2500 kHz. The band structures are derived assuming periodic (in the $x$-direction) and free (in the $y$-direction) boundary conditions at the edges of the cell domains due to the finite dimensions of the considered structures. The resulting eigenvalue problem $(\mathbf{K} - \omega^2\mathbf{M})\mathbf{u} = \mathbf{0}$ is solved by varying the wave vector $k_x$ values along the first irreducible Brillouin zone boundary $\Gamma - X$ (since the periodicity is only in the $x$-direction).

### A. Dispersion diagrams of the A1, A2 and A3 regions

Figure SM3 presents the band diagram for the unit cells composing regions A1, A2 and A3 in terms of reduced wavevector $k^* = k_x \cdot ai/\pi$ with $ai$ given in Table I. It is possible to see how the region A1 (Fig. SM3a) provides filtering lower frequencies ($f < f_1$) whereas regions A2 and A3 (Fig. SM3b,c) filter $f > f_1$ (BGs are highlighted by light pink rectangles).

### B. Dispersion diagrams of the FB2

Figure SM4 presents the band diagram for FB2 in terms of reduced wavevector $k^* = k_x \cdot A/\pi$ with $A$ given in Table II. It is possible to see how FB2 provides filtering for all

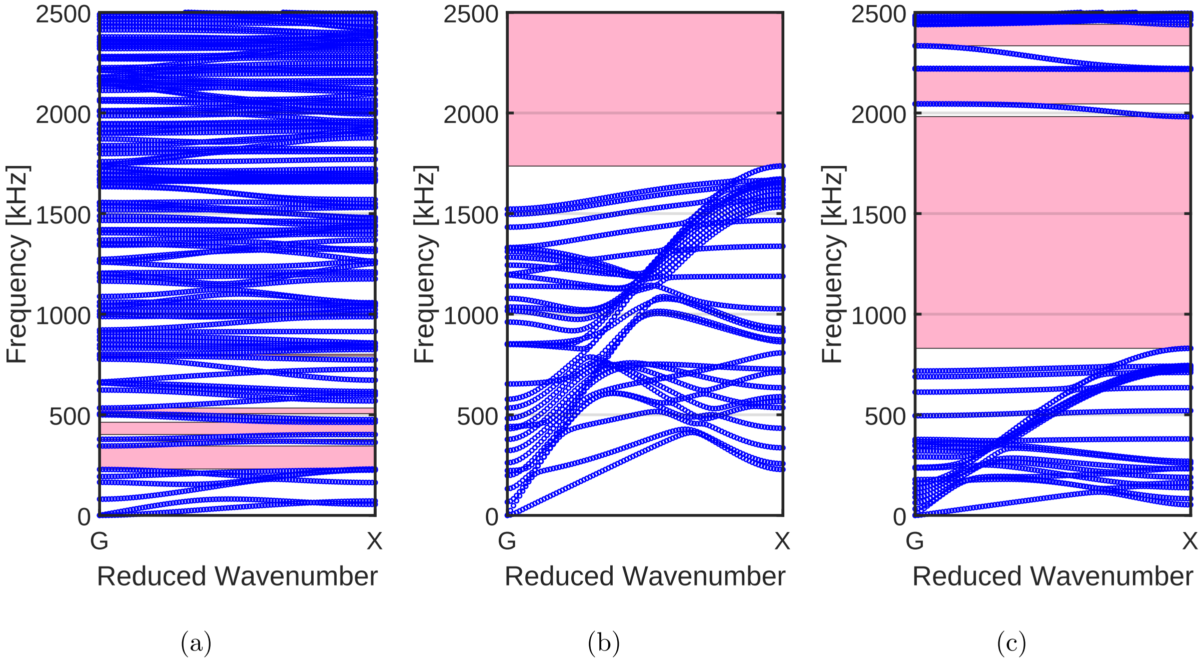


FIG. SM3: Numerically-predicted dispersion band structure for the (a) A1, (b) A2 and (c) A3 regions.

the waves with $f \geq f_1$ allowing on the other hand $f_0$ to pass through the barrier (BGs are highlighted by light pink rectangles).

### C. Propagation LtR and RtL

With the design described above we performed a Left to Right (LtR) and Right to Left (RtL) propagation simulation experiment. In Fig. SM5 the spectral analysis of the signals for LtR (a-d) and RtL (e-h) propagation are reported, respectively. With respect to Fig. 2 of the main text we added the FFT magnitude in the right cavity. As can be noted the diode functionality is confirmed by the fact that the component $f_1$ propagates from LtR, while no signal is detected at the receiver when the propagation is in the other direction.

## III. DISCUSSION ON THE EFFICIENCY OF THE DIODE

We performed an additional analysis on the efficiency of the presented device. The design, the choice of the intensity of the nonlinear source, the dimension of the three different zones

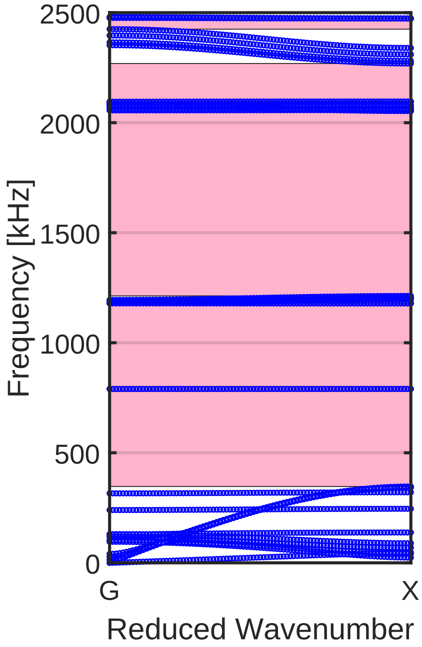


FIG. SM4: Dispersion diagram for the FB2.

of the device can change and significantly influence the results, due to the possibility to exploit resonances of the sample and to optimize the characteristics of the nonlinearity (position, form, excitation, etc.). Here, we demonstrate how the efficiency of the device can be tuned and evaluate its dependence on the main parameters.

Taking for simplicity a quadratic nonlinearity, as stated in the main text, the generation of the frequency mixing in the sidebands is proportional to the product of the amplitudes of the two mixed frequency sources ($A_1$ and $A_2$ for sources $S_1$ and $S_2$, respectively), through the nonlinear parameter ($\beta_1$). Then, we expect the amplitude of the sub-harmonic, $f_0$, to be

$$A_0 \propto \beta_1 A_1 A_2 \tag{SM1}$$

Similarly, for the generation of the harmonics of $f_0$ in the second nonlinear zone (with nonlinear parameter $\beta_2$), we expect the amplitude of the output to be

$$A_{out} \propto \beta_2 A_0^2 = \beta_2 \beta_1^2 A_1^2 A_2^2. \tag{SM2}$$

From Eq. SM2 it is evident that the efficiency, $e$, which can be defined as the ratio between the squared input and output amplitudes, can be approximately estimated as:

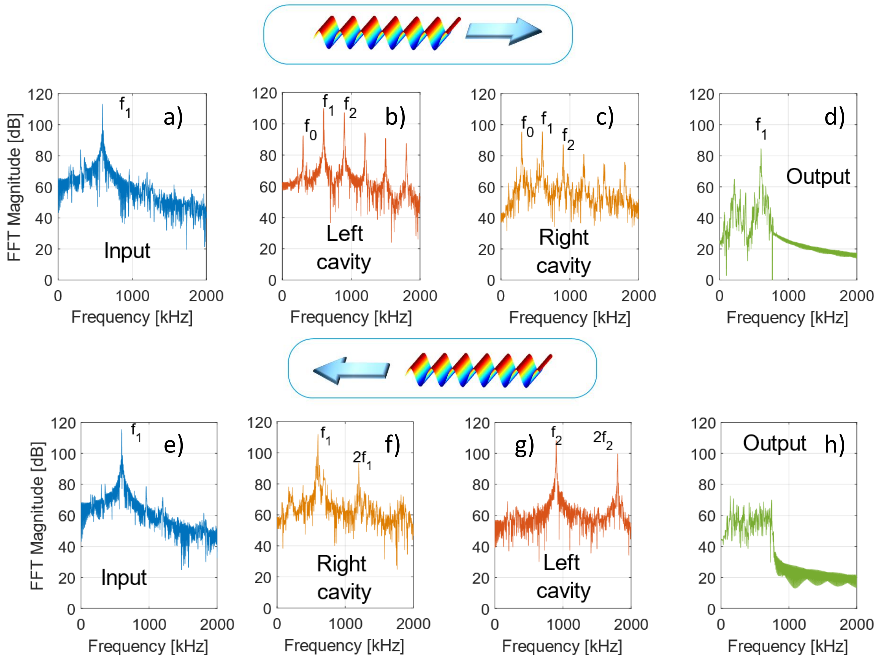


FIG. SM5: Spectral content of the wavefield during Left to Righ (a-d) and Right to Left (e-h) propagation.

$$e = \frac{A_{out}^2}{A_1^2} \propto \beta_2^2 \beta_1^4 A_1^2 A_2^4. \quad \text{(SM3)}$$

To verify this hypothesis, we performed a specific parametric study of a 1-D device working at $f_1 = 1.2\,\text{MHz}$ (so that $f_2 = 1.8\,\text{MHz}$ and $f_0 = 0.6\,\text{MHz}$). The principal propagating medium (the matrix) is an alluminium bar ($\rho_1 = 2700\,\text{kg/m}^3$, $E_1 = 70\,\text{GPa}$), 180 mm in length. The phononic crystal is realized by alternating layers of alluminium and a second material with reduced modulus and density ($\rho_2 = 200\,\text{kg/m}^3$, $E_2 = 7\,\text{GPa}$, e.g. wood). The three filtering barriers are designed by varying the characteristics of the unit cell (size and filling factor) as reported in Table III. FB2, in this case, is realized as a sequence of two phononic crystals (FB2$_A$ and FB2$_B$), in order to maximize the width of the filter and to cut frequencies above $f_0$.

To verify Eq. SM2 and estimate the intensity of the nonlinear parameters and proportionality between the two nonlinear zones, in order to obtain the desirable efficiency in this 1-D model, we performed a series of simulations varying the quadratic nonlinear parameters of

TABLE III: Geometrical parameters for the unit cell of the 1-D model.

| Barrier | Cell size [mm] | Width of the inclusion [mm] | Passing frequency |
|---|---|---|---|
| FB1 | 0.9 | 0.8 | $f_p \leq f_1$ |
| $FB2_A$ | 1.4 | 1.2 | $f_p < f_1$ |
| $FB2_B$ | 0.9 | 0.8 | $f_p < f_2$ |
| FB3 | 2.7 | 2.4 | $f_0 < f_p \leq f_1$ |

the two nonlinear zones ($\beta_1$ and $\beta_2$). As shown in Fig. SM6, simulations highlight the linear and quadratic dependence of the output vs input amplitudes on the nonlinear parameters.

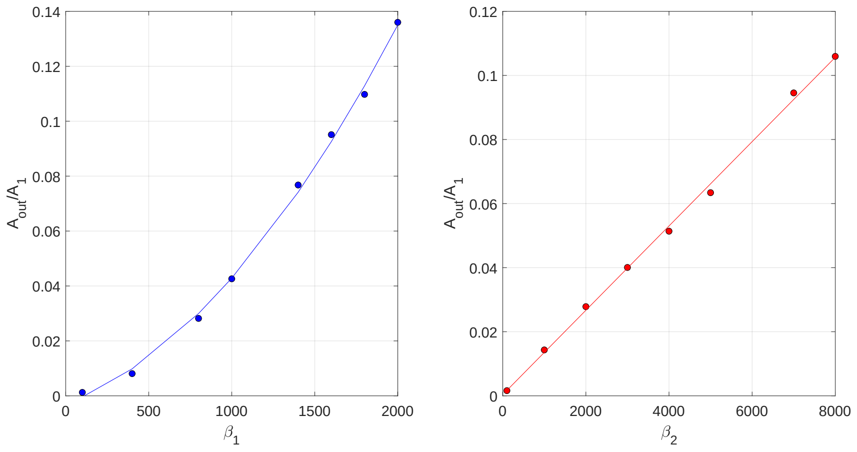


FIG. SM6: Numerical parametric study: (a) Dependence of Output/Input amplitudes as a function of nonlinear parameter $\beta_1$; (b) Dependence of Output/Input amplitudes as a function of nonlinear parameter $\beta_2$.

## IV. SAMPLES AND EXPERIMENTAL CONFIGURATION

A schematic representation of the experimental specimen is shown in Fig. SM7, along with its dimensions (in mm). The specimen consists of a 6 mm-thick aluminum plate with a 2D array of $4 \times 8$ cross-like cavities machined in its middle portion. The sample is obtained

via water-jet cutting starting from a unique pristine aluminum sheet, exhibiting linear elastic properties with the following nominal mechanical parameters $\rho = 2700\,\text{kg/m}^3$, $E = 70\,\text{GPa}$ $\nu = 0.33$.

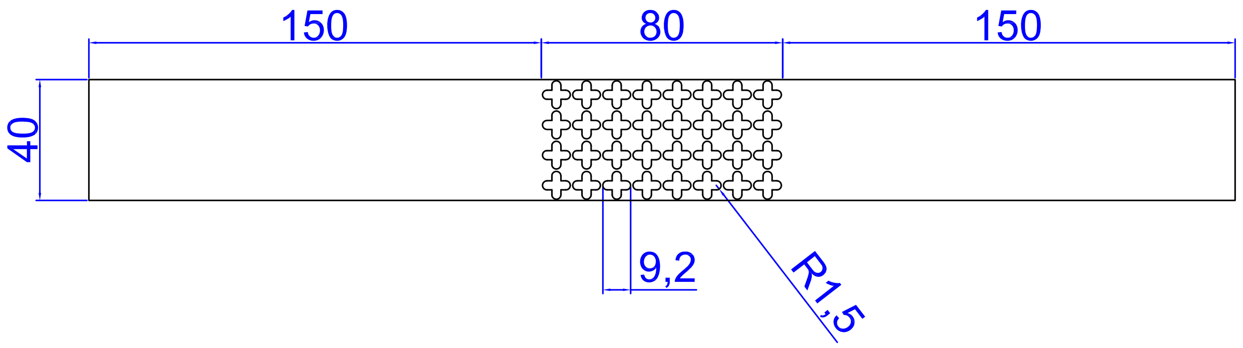


FIG. SM7: Schematic representation of the specimen composed of an aluminium plate with a phononic crystal region made of cross-like holes. Dimensions are in mm. Plate thickness is 6 mm.

The corresponding dispersion diagram of the periodic structure presented in Fig. SM7 is computed numerically applying the Bloch-Floquet periodic boundary conditions, which allow to consider only one unit cell [2]. A full 3D model is implemented to capture all the possible wave modes propagating in the structure. Cells are meshed by means of 4-node tetrahedral elements of maximum size $L_{FE} = 1$ mm in order to provide accurate eigensolutions up to the maximum frequency of interest.

The band structure is derived assuming periodic (in the $x$-direction) and free (in the $y$-direction) boundary conditions at the edges of the cell domain. Free boundary conditions are imposed at the top and bottom surfaces of the cell. The resulting eigenvalue problem $(\mathbf{K} - \omega^2\mathbf{M})\mathbf{u} = \mathbf{0}$ is solved by varying the wave vector $\vec{k} = \{k_x, k_y\}$ values along the first irreducible Brillouin zone boundary $\Gamma - X$ (since the periodicity is only in the $x$-direction).

Figure SM8 presents the band structures in terms of reduced wavevector $k^* = [k_x \cdot a/\pi$ with $a = 10$ mm (lattice geometrical parameters are given in mm in Fig. SM7). Two main BGs (light pink rectangles) exist in the $[0 - 250]$ kHz frequency range, with the lower one extending from 124 kHz to 175 kHz. The other extends from 191 kHz to 236 kHz, apart from very flat bands ranging from 201 kHz to 203 kHz. The experimental results are in excelent agreement with the numerical predictions (see Fig.SM8b).

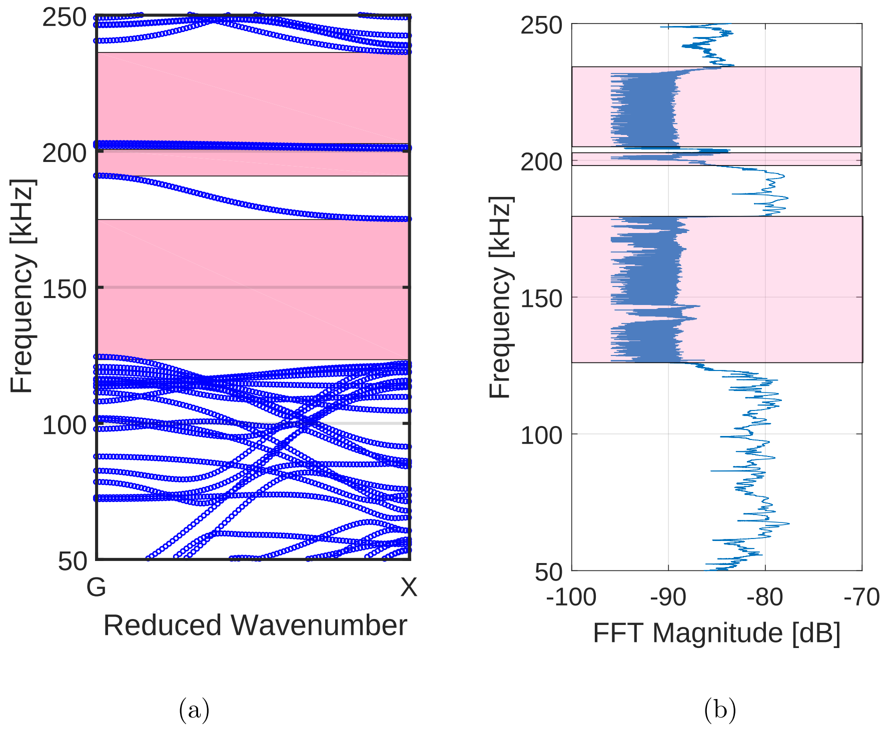


FIG. SM8: Dispersion diagram for the unit cell used for the experimental investigation (a). FFT of the signal obtained with a propagation experiment.

## V. EXPERIMENTAL RESULTS

To verify the functionality of the experimental set-up, we study here the spectral content of the signal generated by the transducer $S1$ (Fig.SM9a), as it travels through the metastructure. Figs. SM9(b-d) show the FFT analysis of the wavefield recorded at three different points A1, A2 and A3 in the sample, as detected by the laser vibrometer: only the nonlinearity in the first cavity (NL1) is activated (no reciprocity breaking is expected in this case), so the only frequency that can travel from left to right is the frequency $f_0 = 75\,\text{kHz}$, generated by wave mixing in the left cavity. The small peak occurring at 450 kHz corresponds to the second harmonic of $f_2$, which is not filtered by the phononic crystal.

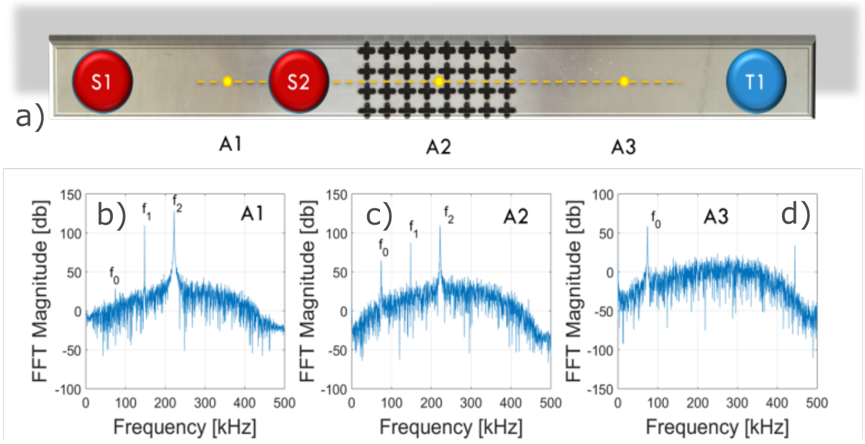


FIG. SM9: (a) Experimental device and setup. (b-d) Spectral content of the signal recorded at three different points A1, A2 and A3 in the sample, as detected by the laser vibrometer along the dashed line schematic (a).

---


[1] M. Miniaci, M. Mazzotti, M. Radzieński, N. Kherraz, P. Kudela, W. Ostachowicz, B. Morvan, F. Bosia, N. M. Pugno. Experimental Observation of a Large Low-Frequency Band Gap in a Polymer Waveguide, Front. Mater., **5**, 2018.

[2] M. Collet, M. Ouisse, M. Ruzzene, and M. Ichchou, Int. J. Solids Struct. 48, 2837 (2011).
